# Long-term pulmonary function after intrathoracic versus extrathoracic LVAD Implantation: perioperative implications at one year

**DOI:** 10.1186/s40981-026-00859-3

**Published:** 2026-05-02

**Authors:** Kimito Minami, Tatsutoshi Shimatani, Masahiro Kazawa, Naoki Tadokoro, Satsuki Fukushima, Takuma Sato, Yasumasa Tsukamoto, Muneyuki Takeuchi

**Affiliations:** 1 Department of Critical Care Medicine, National Cerebral and Cardiovascular Center, 6-1 Kishibeshinmachi, Suita, Osaka 564-8565 Japan; 2 Department of Cardiac Surgery, National Cerebral and Cardiovascular Center, 6-1 Kishibeshinmachi, Suita, Osaka 564-8565 Japan; 3 Department of Transplant Medicine, National Cerebral and Cardiovascular Center, 6-1 Kishibeshinmachi, Suita, Osaka 564-8565 Japan

**Keywords:** Left ventricular assist device, HeartMate 3, HeartMate II, Pulmonary function, Forced vital capacity, Restrictive ventilatory defect, Mechanical circulatory support

## Abstract

**Background:**

Patients undergoing durable left ventricular assist device (LVAD) implantation frequently require prolonged perioperative respiratory support. However, the long-term pulmonary consequences of different surgical pump placement strategies remain unclear. We investigated longitudinal pulmonary function changes following intrathoracic versus extrathoracic LVAD implantation.

**Methods:**

This single-center retrospective cohort study included patients who underwent LVAD implantation and completed pulmonary function testing (PFT) preoperatively and at 1 year postoperatively. Intrathoracic HeartMate 3 recipients were compared with extrathoracic HeartMate II recipients. Linear mixed-effects models assessed longitudinal changes in forced vital capacity (FVC), percent predicted FVC (%FVC), forced expiratory volume in 1 s (FEV₁), and percent predicted FEV₁ (%FEV₁), adjusting for age, sex, body surface area, and surgery era.

**Results:**

Among 190 patients (intrathoracic, *n* = 76; extrathoracic, *n* = 114), greater declines in FVC, %FVC, and FEV₁ were observed at 1 year in the intrathoracic group. Significant time-by-placement interactions were identified for FVC (*P* = 0.010), %FVC (*P* = 0.010), and FEV₁ (*P* = 0.011), whereas %FEV₁ showed comparable trends between groups. Smaller body surface area and female sex were independently associated with lower lung volumes, whereas the apparent association with female sex was largely explained by body size.

**Conclusions:**

Intrathoracic LVAD implantation was associated with greater restrictive changes in pulmonary function at one year. These findings suggest that surgical pump positioning and patient body size may influence long-term respiratory trajectories and may be relevant for perioperative planning and postoperative follow-up.

**Supplementary Information:**

The online version contains supplementary material available at 10.1186/s40981-026-00859-3.

## Introduction

Implantable left ventricular assist devices (LVADs) have become a cornerstone of durable mechanical circulatory support for patients with advanced heart failure [1]. While contemporary LVAD development has focused on improving hemocompatibility and durability, the mechanical interaction between implanted devices and surrounding thoracic structures has received comparatively less attention. Most current and next-generation LVADs adopt an intrathoracic pump configuration, placing the device in close proximity to the lung and diaphragm. Such anatomical positioning may impose space-occupying constraints that influence respiratory mechanics [2, 3]. 

Earlier-generation devices utilizing extrathoracic or preperitoneal placement offer a unique opportunity to examine configuration-related mechanical effects on pulmonary function. In this context, the HeartMate II (Abbott, Abbott Park, IL, USA), which requires creation of a preperitoneal pump pocket, serves as a historical reference model to contrast intrathoracic LVAD configuration represented by the HeartMate 3 (Abbott, Abbott Park, IL, USA), rather than as a comparator of contemporary device performance. However, data directly comparing pulmonary outcomes between intrathoracic and extrathoracic LVAD configurations remain limited, and no study has systematically evaluated longitudinal changes in standardized pulmonary function tests (PFTs) across these cohorts.

Therefore, we conducted a retrospective observational study to compare preoperative and 1-year postoperative PFTs in HeartMate 3 and HeartMate II recipients. We hypothesized that anatomical differences in pump placement would result in distinct postoperative trajectories of lung function, with the intrathoracic HeartMate 3 potentially exerting a greater restrictive effect.

## Materials and methods

### Ethical considerations

This study was approved by the institutional ethics committee (registration number: R23008). Given the retrospective design and absence of invasive interventions, the requirement for informed consent was waived. No external funding was received for the conduct of this study. No Artificial Intelligence–generated content tools were used in the development, writing, or editing of any portion of this manuscript.

### Study design and population

This was a single-center, retrospective observational study conducted at a tertiary cardiovascular institution. Patients who underwent LVAD implantation between April 2010 and May 2023 were screened for eligibility. Inclusion criteria were: [1] survival to 1 year after LVAD implantation, and [2] availability of pulmonary function test data both preoperatively and at 1 year postoperatively. Patients meeting these criteria were selected for analysis. In addition to HeartMate 3 and HeartMate II devices, the HeartWare HVAD (Medtronic, Minneapolis, MN, USA) was also available as an LVAD option during a limited period in Japan. However, because the HeartWare HVAD was approved for only a short interval and subsequently withdrawn from the market, the number of implanted cases at our institution was extremely small. As a result, meaningful statistical analysis of postoperative pulmonary function in HeartWare HVAD recipients was not feasible, and these patients were therefore excluded from the present study.

### Outcome measures

Patients were categorized according to LVAD pump configuration as either an intrathoracic configuration group (HeartMate 3) or an extrathoracic configuration group (HeartMate II). The primary objective was to examine the interaction between pump configuration (intrathoracic or extrathoracic) and time (pre- vs. 1 year post-implantation) on pulmonary function outcomes. The specific pulmonary function test parameters analyzed included FVC, percent predicted FVC (%FVC), FEV_1_, and percent predicted FEV_1_ (%FEV_1_). Pulmonary function data and patient background characteristics were extracted from electronic medical records.

### Sample size

Formal sample size calculation for linear mixed-effects models is challenging because required sample size depends on several unknown factors, including the within-subject correlation structure and variability across time points. Therefore, as commonly done in clinical studies employing mixed-effects modeling, sample size adequacy was evaluated based on the number of participants relative to the fixed-effect parameters and the availability of repeated measurements. In the primary mixed-effects models, 190 patients contributed repeated measurements at two prespecified time points (pre-surgery and 1-year follow-up). Given the limited number of fixed-effect parameters (time, configuration type, their interaction, and four covariates described below) and the availability of two observations per patient, this sample size was considered adequate to ensure stable estimation of model parameters and reliable inference for both main effects and the time-by- configuration interaction.

### Statistical analysis

Pulmonary function outcomes (FVC, %FVC, FEV_1_, and %FEV_1_) were evaluated using linear mixed-effects models to compare early postoperative changes between extrathoracic and intrathoracic LVAD configurations. Because follow-up measurements beyond the first postoperative year were sparse and highly unbalanced, the primary analysis was restricted to two prespecified time points: pre-surgery and 1 year post-surgery. To inform covariate selection, a directed acyclic graph was constructed to represent the assumed relationships among LVAD configuration, temporal factors, patient characteristics, and pulmonary function outcomes. Based on this framework, age, sex, body surface area, and surgical era were identified as potential confounders and included in the primary models. (The directed acyclic graph is presented in Supplementary Figure S1.) For each outcome, a mixed-effects model including time, pump configuration (intrathoracic vs. extrathoracic), and their interaction term was fitted. The primary models adjusted for age, sex, body surface area, and surgical era to account for potential temporal confounding. A subject-specific random intercept accounted for within-patient correlation. Preoperative pulmonary function values were not included as covariates in the primary models because they are part of the longitudinal outcome vector and were therefore not treated as confounders. Instead, baseline-adjusted models were performed as supplementary analyses. (Supplementary Figure S2.) Models were fitted using the lme4 and lmerTest packages, and p-values for the time effect and the time×configuration interaction were obtained from lmerTest. To present adjusted results, we estimated marginal means for each configuration group at both time points using the emmeans package, along with 95% confidence intervals. Group differences (intrathoracic – extrathoracic) at each time point were summarized using pairwise contrasts, displayed as forest plots. The four pulmonary function outcomes were displayed in 2 × 2 panels for both adjusted marginal means plots and forest plots. Each marginal means subplot included the corresponding P for time and P for interaction. Main effects (time, configuration type and time×configuration interaction) were interpreted using the conventional two-sided α = 0.05 threshold. Continuous variables were summarized as medians with interquartile ranges, while categorical variables were reported as frequencies and percentages. Statistical analyses were performed using R version 4.0.3 (R Foundation for Statistical Computing, Vienna, Austria; www.R-project.org).

## Results

### Patient characteristics

A total of 230 patients underwent LVAD implantation during the study period. Of these, 190 patients met the inclusion criteria, having completed both preoperative and 1-year postoperative pulmonary function testing. The cohort comprised 76 patients who received the intrathoracic configuration and 114 patients who received the extrathoracic configuration. Baseline demographic data and preoperative clinical characteristics for the full cohort are summarized in Table 1.

**Table 1:** Patient characteristics by configuration type

	Overall	Intrathoracic (HM3)	Extrathoracic (HMII)	*P*
Number	190	76	114	
Age, years	47 [37, 57]	50 [40, 59]	45 [36, 55]	0.093
Female sex, n (%)	58 (30.5%)	25 (32.9%)	33 (28.9%)	0.563
Height, cm	166.9 [159.9, 172.8]	166.1 [159.0, 172.4]	167.7 [160.5, 173.0]	0.537
Weight, kg	56.6 [48.0, 69.0]	55.4 [46.1, 68.1]	57.9 [48.7, 69.5]	0.196
Body surface area, m^2^	1.64 [1.48, 1.81]	1.62 [1.44, 1.78]	1.65 [1.52, 1.83]	0.200
Underlying disease, n (%)				0.700
DCM	91 (47.9%)	35 (46.1%)	56 (49.1%)	
ICM	23 (12.1%)	11 (14.5%)	12 (10.5%)	
HCM	27 (14.2%)	12 (15.8%)	15 (13.2%)	
Myocarditis	9 (4.7%)	3 (3.9%)	6 (5.3%)	
Others	40 (21.1%)	15 (19.7%)	25 (21.9%)	
Surgical era	2018 [2016, 2020]	2021 [2020, 2022]	2017 [2015, 2017]	< 0.001
Hypertension, n (%)	11 (5.8%)	1 (1.3%)	10 (8.8%)	1.000
Preoperative Alb, g/dL	3.7 [3.2, 4.0]	3.6 [3.3, 4.0]	3.7 [3.2, 4.0]	0.740
Preoperative Cr, mg/dL	0.96 [0.81, 1.24]	0.96 [0.76, 1.22]	0.96 [0.82, 1.29]	0.596
Preoperative BUN, mg/dL	18 [14, 24]	18 [14, 22]	19 [14, 26]	0.227
Preoperative T-Bil, mg/dL	0.9 [0.6, 1.4]	0.9 [0.7, 1.3]	0.9 [0.6, 1.7]	0.503
Preoperative BNP, pg/dL	579 [299, 1095]	604 [293, 1160]	575 [338, 1038]	0.950
Diabetes, n (%)	34 (17.9%)	3 (3.9%)	31 (27.2%)	0.344
Smoking history, n (%)	61 (32.1%)	4 (5.3%)	57 (50.0%)	0.172
Preoperative FVC, L	3.02 [2.40, 3.46]	2.90 [2.22, 3.32]	3.07 [2.52, 3.49]	0.066
Preoperative %FVC	88.0 [75.5, 99.5]	84.7 [75.9, 97.4]	90.4 [75.5, 102.6]	0.266
Preoperative FEV_1_, L	2.35 [1.86, 2.83]	2.16 [1.64, 2.74]	2.39 [1.97, 2.84]	0.030
Preoperative %FEV_1_	79.0 [74.3, 83.8]	78.0 [73.4, 82.3]	80.0 [76.2, 84.0]	0.113

*Alb* albumin, *BNP* B-type natriuretic peptide, *BUN* blood urea nitrogen, *Cr* creatinine, *DCM* dilated cardiomyopathy, *FEV1* forced expiratory volume in one second, *FVC* forced vital capacity, *HCM* hypertrophic cardiomyopathy, *ICM* ischemic cardiomyopathy, *T-Bil* total bilirubin, *%FEV1* percent predicted forced expiratory volume in one second, *%FVC* percent predicted forced vital capacity.

Data are presented as n (%) or median [interquartile range]

## Primary outcomes

**Fig. 1:**
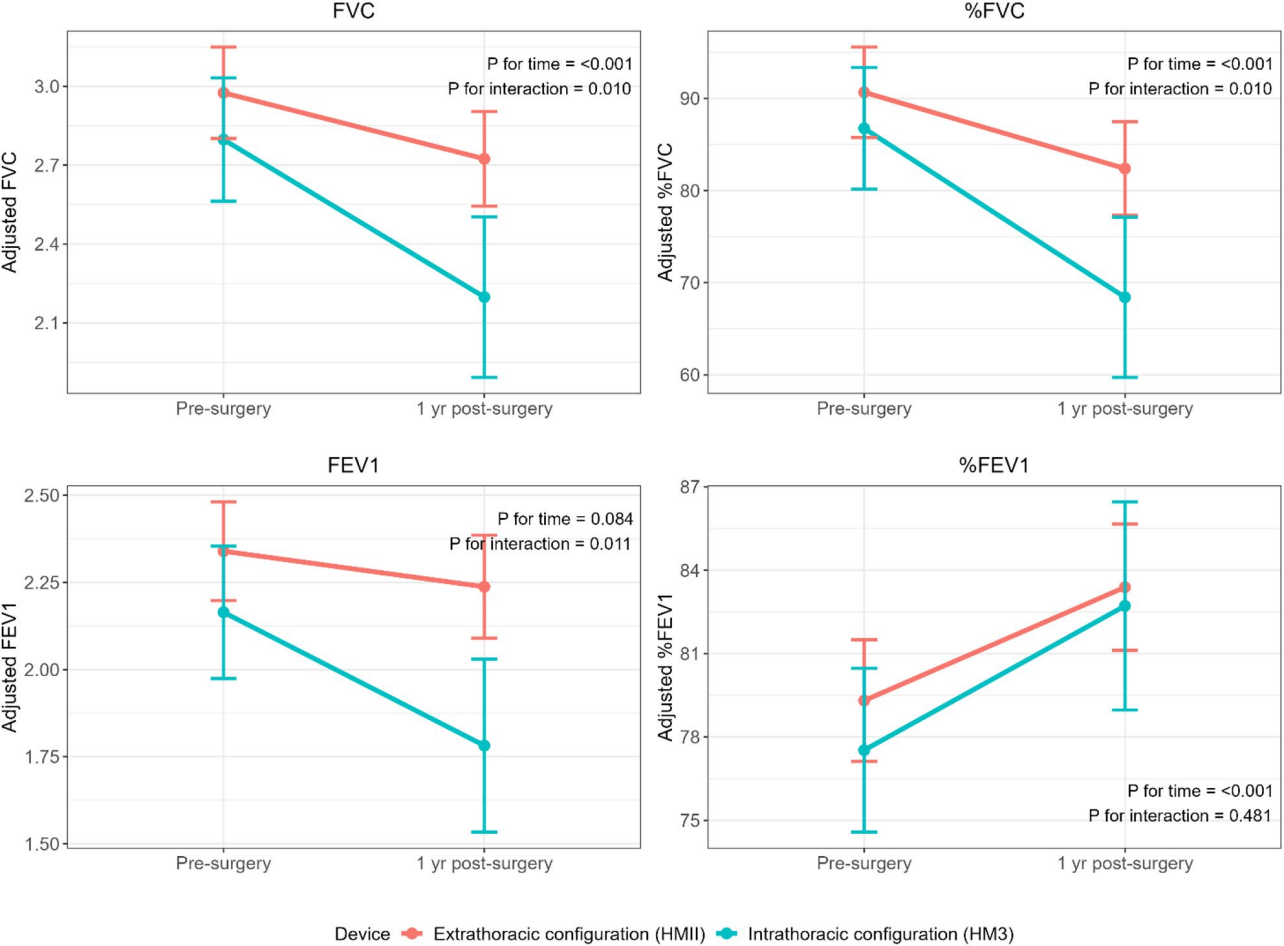
Adjusted marginal means (95% confidence intervals) for each pulmonary function outcome at pre-surgery and 1 year post-surgery in the intrathoracic and extrathoracic configuration recipients. Estimates were derived from linear mixed-effects models including time, configuration type, their interaction, and covariates (age, sex, body surface area, and surgical era), with a random intercept for subjects. P-values and interaction P-values shown in each panel correspond to the fixed effects for time and the time × configuration interaction

**Fig. 2:**
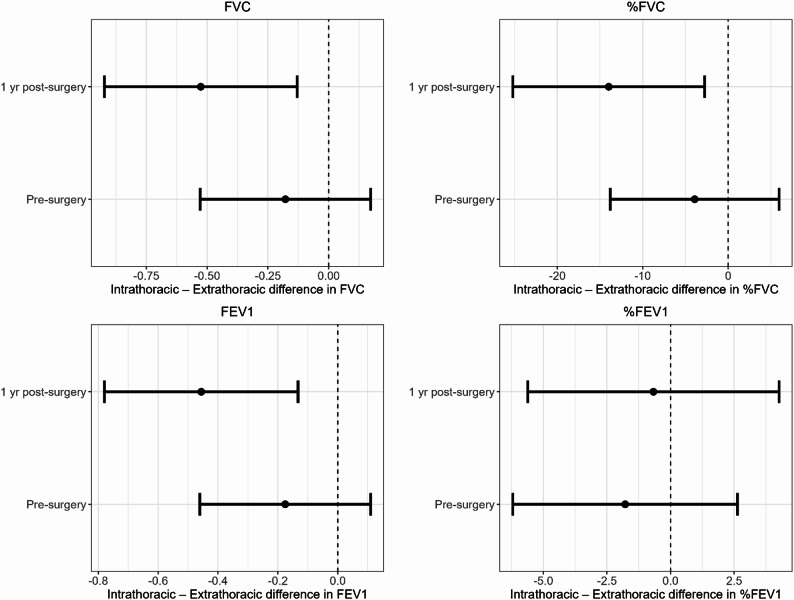
Forest plots illustrating adjusted between-configuration differences (intrathoracic – extrathoracic) in pulmonary function outcomes at pre-surgery and at 1 year post-surgery. Estimates and 95% confidence intervals were derived from pairwise contrasts of marginal means obtained from mixed-effects models. Negative values indicate lower pulmonary function in the intrathoracic configuration group

 Mixed-effects linear regression analyses demonstrated distinct longitudinal changes in pulmonary function between the two configuration groups. Significant time-by- configuration interactions were observed for FVC (difference in change: -0.35 L, 95% CI -0.61to -0.08; P for interaction = 0.010), %FVC (-10.06%, 95% CI -17.69 to -2.43; P for interaction = 0.010), and FEV1 (-0.28 L, 95% CI -0.50 to -0.06; P for interaction = 0.011), indicating that the decline in lung function from baseline to 1 year postoperatively was more pronounced among patients who received the intrathoracic configuration compared with those who received the extrathoracic configuration. In contrast, the interaction for %FEV1 did not reach statistical significance (difference in change: 1.11%, 95% CI -2.00 to 4.23; P for interaction = 0.481), suggesting a similar temporal pattern of change between configuration types for this parameter. In addition to the interaction effects, adjusted marginal means at each time point and between-group differences are presented in Table 2. Estimated marginal means illustrating the longitudinal changes in pulmonary function by configuration type are shown in Fig. 1. Adjusted between-configuration differences in pulmonary function outcomes at each time point are presented as a forest plot in Fig. 2. In adjusted fixed-effect estimates (Table 3), lower body surface area were consistently associated with lower FVC, %FVC, and FEV₁, indicating that smaller patients experienced greater postoperative restrictive impairment regardless of configuration type.

**Table 2:** Adjusted estimated marginal means from mixed-effects models of pulmonary function outcomes at baseline and 1-year follow-up, configuration-specific changes, and between-configuration differences

Outcome	Device	Pre-surgery	1 year post-surgery	Delta	*P* for interaction
FVC, L	Extrathoracic	2.98 (2.80–3.15)	2.72 (2.54–2.90)	-0.25 (-0.39–-0.11)	
FVC, L	Intrathoracic	2.80 (2.56–3.03)	2.20 (1.89–2.50)	-0.60 (-0.83–-0.37)	
FVC, L	Difference in Δ (Intrathoracic – Extrathoracic)			-0.35 (-0.61–-0.08)	0.010
%FVC	Extrathoracic	90.67 (85.76–95.57)	82.39 (77.31–87.47)	-8.28 (-12.45–-4.11)	
%FVC	Intrathoracic	86.75 (80.15–93.35)	68.41 (59.72–77.10)	-18.34 (-25.03–-11.65)	
%FVC	Difference in Δ (Intrathoracic – Extrathoracic)			-10.06 (-17.69–-2.43)	0.010
FEV_1_, L	Extrathoracic	2.34 (2.20–2.48)	2.24 (2.09–2.39)	-0.10 (-0.22–0.02)	
FEV_1_, L	Intrathoracic	2.16 (1.97–2.35)	1.78 (1.53–2.03)	-0.38 (-0.57–-0.19)	
FEV_1_, L	Difference in Δ (Intrathoracic – Extrathoracic)			-0.28 (-0.50–-0.06)	0.011
% FEV_1_	Extrathoracic	79.31 (77.12–81.49)	83.39 (81.12–85.66)	4.08 (2.39–5.77)	
% FEV_1_	Intrathoracic	77.53 (74.58–80.47)	82.72 (78.97–86.46)	5.19 (2.46–7.93)	
% FEV_1_	Difference in Δ (Intrathoracic – Extrathoracic)			1.11 (-2.00–4.23)	0.481

Adjusted means and 95% confidence intervals were obtained from linear mixed-effects models including time, configuration type, their interaction, and covariates (age, sex, body surface area, and surgical era), with a random intercept for participants

*FVC* forced vital capacity, *%FVC* percent predicted forced vital capacity,_1_*FEV* forced expiratory volume in one second, _1_*%FEV* percent predicted forced expiratory volume in one second

Δ indicates adjusted within-configuration change from pre-surgery to 1 year

The “Difference in Δ (Intrathoracic – Extrathoracic)” reflects the adjusted between-configuration difference in 1-year change

Interaction P-values correspond to the time × configuration term

**Table 3:** Fixed-effect coefficients from mixed-effects models

Outcome	Term	Estimate	SE	95% CI	*P*
FVC	(Intercept)	-13.85	63.70	-139.45–111.75	0.828
FVC	Time (1-year vs. Pre-op)	-0.25	0.07	-0.39 – -0.11	< 0.001
FVC	Configuration type (Intrathoracic vs. Extrathoracic)	-0.18	0.17	-0.52–0.17	0.310
FVC	Age (per year)	0.00	0.00	-0.01–0.00	0.322
FVC	Sex (Female vs. Male)	-0.49	0.12	-0.73 – -0.25	< 0.001
FVC	Body surface area (per m²)	1.53	0.27	1.00–2.05	< 0.001
FVC	Surgical era	0.01	0.03	-0.06–0.07	0.818
FVC	Interaction: Time × Configuration	-0.35	0.13	-0.61 – -0.08	0.010
%FVC	(Intercept)	-202.08	1792.21	-3735.63–3331.48	0.910
%FVC	Time (1-year vs. Pre-op)	-8.28	2.06	-12.37 – -4.19	< 0.001
%FVC	Configuration type (Intrathoracic vs. Extrathoracic)	-3.92	4.92	-13.62–5.78	0.427
%FVC	Age (per year)	0.25	0.09	0.07–0.43	0.006
%FVC	Sex (Female vs. Male)	8.64	3.41	1.91–15.36	0.012
%FVC	Body surface area (per m²)	23.12	7.48	8.37 − 37.86	0.002
%FVC	Surgical era	0.12	0.89	-1.64–1.87	0.894
%FVC	Interaction: Time × Configuration	-10.06	3.86	-17.69 – -2.43	0.010
FEV1	(Intercept)	-17.10	51.78	-119.20–85.00	0.742
FEV1	Time (1-year vs. Pre-op)	-0.10	0.06	-0.22–0.01	0.084
FEV1	Configuration type (Intrathoracic vs. Extrathoracic)	-0.18	0.14	-0.46–0.11	0.219
FEV1	Age (per year)	-0.01	0.00	-0.01 – -0.00	0.005
FEV1	Sex (Female vs. Male)	-0.48	0.10	-0.68 – -0.29	< 0.001
FEV1	Body surface area (per m²)	0.85	0.22	0.42–1.27	< 0.001
FEV1	Surgical era	0.01	0.03	-0.04–0.06	0.720
FEV1	Interaction: Time × Configuration	-0.28	0.11	-0.50 – -0.07	0.011
%FEV1	(Intercept)	-207.66	802.94	-1790.97–1375.65	0.796
%FEV1	Time (1-year vs. Pre-op)	4.08	0.83	2.43–5.73	< 0.001
%FEV1	Configuration type (Intrathoracic vs. Extrathoracic)	-1.78	2.20	-6.12–2.55	0.418
%FEV1	Age (per year)	-0.18	0.04	-0.26 – -0.10	< 0.001
%FEV1	Sex (Female vs. Male)	-3.02	1.53	-6.04–0.00	0.050
%FEV1	Body surface area (per m²)	-13.44	3.33	-20.00 – -6.87	< 0.001
%FEV1	Surgical era	0.16	0.40	-0.63–0.94	0.692
%FEV1	Interaction: Time × Configuration	1.11	1.57	-2.00–4.23	0.481

*FVC* forced vital capacity, *%FVC* percent predicted forced vital capacity,_1_*FEV* forced expiratory volume in one second, _1_*%FEV* percent predicted forced expiratory volume in one second

## Discussion

This study demonstrated that LVAD implantation was associated with significant postoperative pulmonary function decline, which was more pronounced in recipients of intrathoracic configurations than in those with extrathoracic configurations. The differential trajectories observed between configurations suggest that anatomical configuration may contribute to differences in postoperative respiratory mechanics.

The greater reductions in FVC and %FVC observed in intrathoracic configuration recipients appear paradoxical when considering surgical technique alone. The extrathoracic configuration, represented by the HeartMate II, requires creation of a preperitoneal pump pocket and often involves diaphragmatic dissection [4], whereas the intrathoracic configuration, represented by the HeartMate 3, is implanted entirely within the thoracic cavity and generally entails less direct diaphragmatic manipulation. Despite this, our findings suggest that intrathoracic configuration may impose a greater mechanical burden on lung volumes than subdiaphragmatic placement. This aligns with physiological observations seen in unilateral diaphragmatic dysfunction, where lung expansion is mechanically restricted and both FVC and FEV₁ decrease [5–7]. Positioned in close proximity to the left lung, an intrathoracic pump may exert a similar space-occupying structure within the left hemithorax, particularly in patients with limited thoracic reserve. Importantly, the comparison between intrathoracic and extrathoracic configurations inherently reflects differences not only in pump position but also in device generation, implantation era, and perioperative management. Although we adjusted for surgery year in the primary analysis to account for temporal confounding, residual confounding related to changes in patient selection, surgical techniques, and perioperative care cannot be fully excluded. Therefore, the observed associations should not be interpreted as purely reflecting the mechanical effect of pump location alone. In line with this interpretation, smaller body size was a strong and independent predictor of lower postoperative pulmonary function, with lower body surface area consistently associated with lower FVC, %FVC, and FEV₁ across all adjusted models. Although female sex has been linked to smaller thoracic dimensions in prior studies [8], in our analysis, sex was associated with lower adjusted pulmonary function values, but this association appeared to be largely explained by body size, as reflected by body surface area. This likely reflects collinearity between sex and body size. Importantly, body surface area may more directly capture individual thoracic spatial constraints relevant to intrathoracic pump placement, providing a more physiologically meaningful metric than sex alone.

In addition to the observed decline in absolute lung volumes, the pattern of spirometric indices provides further insight into the underlying physiology. The increase in %FEV₁ observed in both groups is consistent with a restrictive pathophysiology. This pattern, characterized by a disproportionate loss of FVC compared with FEV₁, is well described in restrictive ventilatory defects and reflects reduced lung volume rather than improved airflow [9–11]. Thus, the combination of declining absolute volumes and rising %FEV₁ strongly suggests that LVAD implantation, especially with an intrathoracic pump, contributes to mechanical lung restriction rather than airway obstruction.

These findings also contextualize observations from the MOMENTUM 3 trial, which reported a nonsignificant trend toward higher rates of respiratory complications [12, 13]. Although causal interpretation is limited, the anatomical and functional considerations presented in our analysis offer a physiologic rationale for such clinical patterns. Taken together, our results underscore the importance of considering patient-specific thoracic dimensions when selecting LVAD configurations and highlight the need for targeted postoperative pulmonary surveillance in smaller patients, particularly those receiving an intrathoracic pump.

From a perioperative perspective, restrictive ventilatory changes following LVAD implantation may influence postoperative respiratory management, ventilator weaning strategies, and long-term functional recovery. However, because the present study did not directly assess perioperative outcomes such as duration of mechanical ventilation, reintubation, or ICU stay, these interpretations should be considered hypothesis-generating rather than definitive evidence of perioperative impact. Taken together, our findings highlight the potential importance of considering patient-specific thoracic dimensions when selecting LVAD configurations and underscore the need for further studies integrating anatomical, physiological, and clinical outcome data to better understand the respiratory consequences of LVAD implantation.

### Limitation

This study has several limitations. First, its retrospective single-center design introduces the potential for selection bias and limits the generalizability of the findings to other institutions or patient populations. Second, although the mixed-effects models adjusted for major demographic and physiological covariates, unmeasured factors, such as subclinical pulmonary disease or variations in perioperative respiratory management, could not be accounted for and may have influenced postoperative pulmonary trajectories. However, the impact of these omissions may be mitigated by Japan’s rigorous LVAD eligibility criteria. In addition to meeting the criteria for end-stage heart failure, candidates must demonstrate preserved respiratory mechanics, acceptable pulmonary-vascular resistance following vasodilator challenge, an absence of significant smoking history, and adequate functional capacity for self-care [14, 15]. Failure to meet any of these non-cardiac requirements results in exclusion from candidacy. Third, the analysis was restricted to two prespecified time points, as longer-term follow-up data were sparse and highly unbalanced; thus, the temporal evolution of pulmonary function beyond 1 year remains unknown. Fourth, although pulmonary function testing was conducted according to institutional standards, measurement variability inherent to clinical spirometry may have contributed to noise in the observed changes. Fifth, configuration-specific surgical techniques and institutional preferences, including decisions regarding diaphragmatic manipulation and pump positioning, may differ across centers and could influence postoperative pulmonary mechanics. Sixth, the analysis included only patients who survived 1 year and underwent pulmonary function testing, which may introduce survivor bias and limit the generalizability of the findings to more severely ill populations. Finally, differences in implantation era and perioperative management strategies between device groups may have influenced the observed associations. While these limitations warrant cautious interpretation, the consistency of findings across multiple pulmonary parameters supports the robustness of the primary observations.

## Conclusion

In conclusion, LVAD implantation was associated with a significant restrictive decline in pulmonary function at one year, with recipients of intrathoracic devices demonstrating consistently greater reductions than those implanted with extrathoracic devices. These findings suggest that intrathoracic pump placement may exert a disproportionate mechanical effect on lung expansion, particularly in smaller patients. While causal interpretation is limited, consideration of thoracic anatomical constraints may help inform perioperative management and postoperative follow-up. These findings may provide a rationale for considering thoracic anatomical constraints in future studies evaluating LVAD strategies.

## Supplementary Information

Supplementary Material 1.

Supplementary Material 2.

## Data Availability

The datasets generated and analyzed during the current study are available from the corresponding author on reasonable request. All data will be shared in a de-identified format to protect participant confidentiality.
