# Supplementary material for: Long-term pulmonary function after intrathoracic versus extrathoracic LVAD Implantation: perioperative implications at one year
Source: JA Clin Rep. 2026 May 2;12:25. doi: 10.1186/s40981-026-00859-3 (PMC13287512; doi:10.1186/s40981-026-00859-3)
Supplement: Supplementary file 2 — Supplementary Material 2. [file 40981_2026_859_MOESM2_ESM.docx]

**Supplementary Figure S1.**


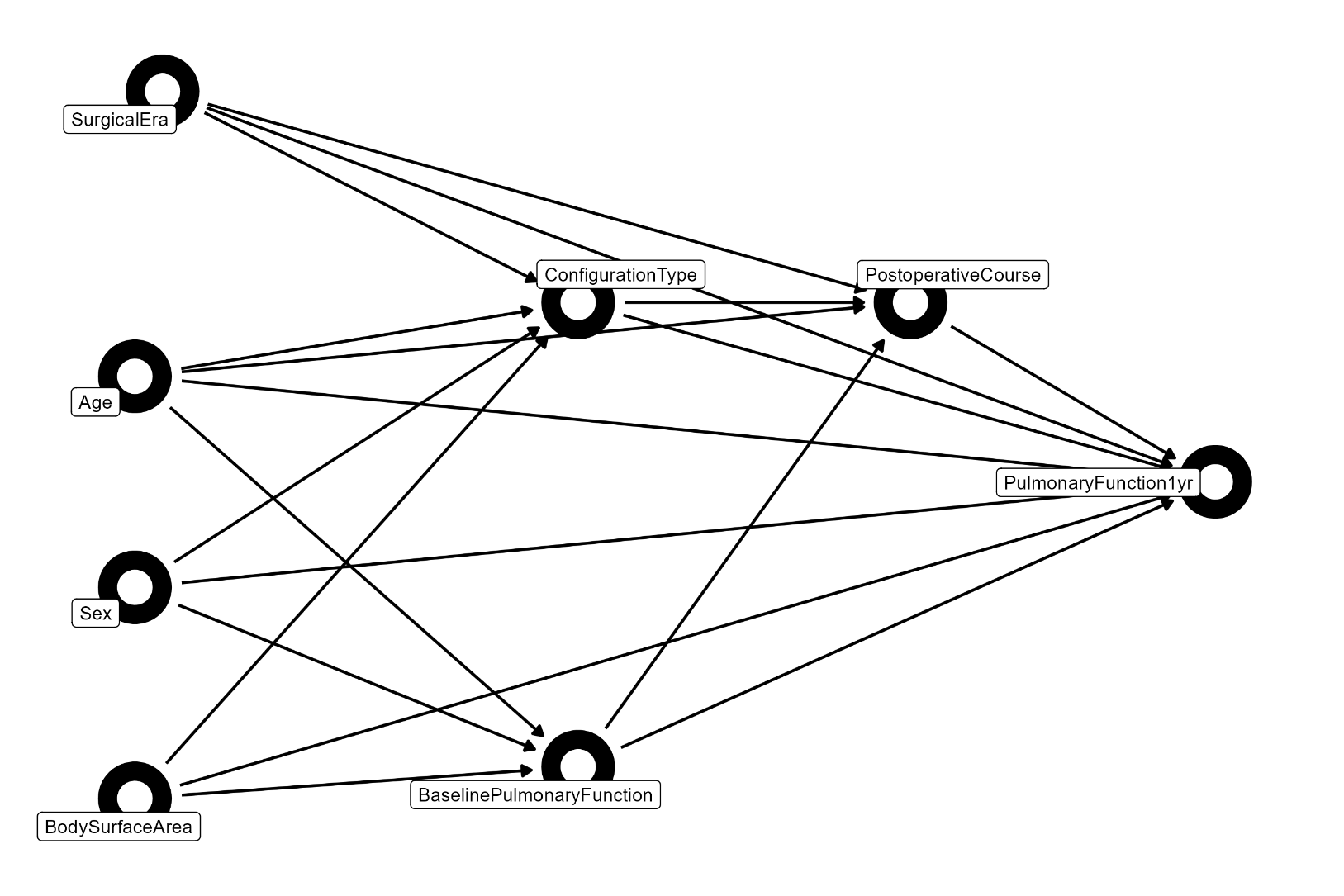


Directed acyclic graph illustrating the assumed causal structure between LVAD configuration and pulmonary function at 1 year. Age, sex, body surface area, and surgical era were considered confounders. Baseline pulmonary function was included as a predictor but not treated as a confounder. Postoperative course represents unmeasured postoperative processes (e.g., complications and recovery trajectory) and was considered a mediator.

**Supplementary Figure S2.**


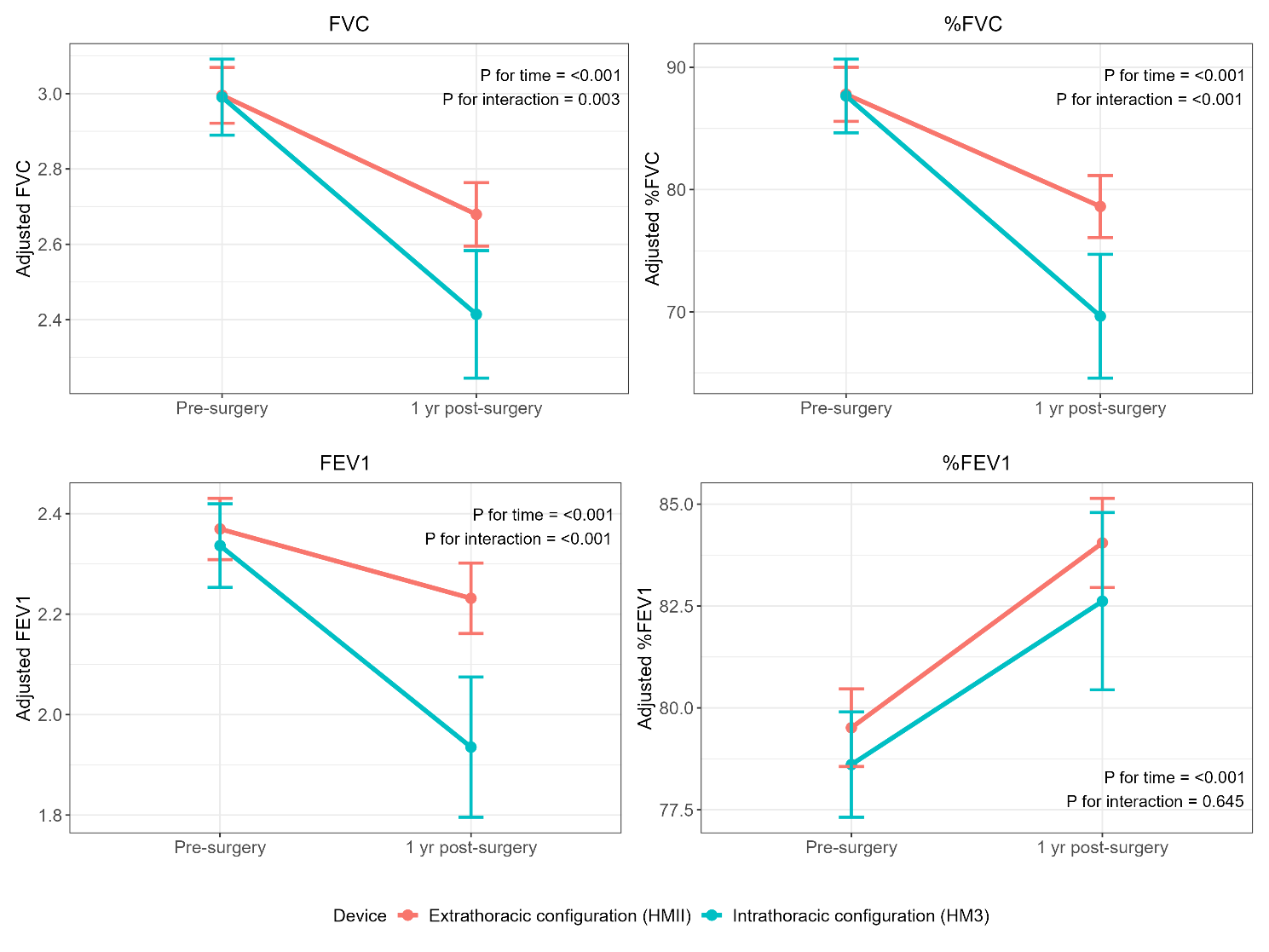


Adjusted marginal means (95% confidence intervals) for each pulmonary function outcome at pre-surgery and 1 year post-surgery in the intrathoracic and extrathoracic configuration recipients. Estimates were derived from linear mixed-effects models including time, configuration type, their interaction, and covariates (age, sex, body surface area, baseline pulmonary function, and surgical era), with a random intercept for subjects. P-values and interaction P-values shown in each panel correspond to the fixed effects for time and the time × configuration interaction.
