# Supplementary material for: Long-term pulmonary function after intrathoracic versus extrathoracic LVAD Implantation: perioperative implications at one year
Source: JA Clin Rep. 2026 May 2;12:25. doi: 10.1186/s40981-026-00859-3 (PMC13287512; doi:10.1186/s40981-026-00859-3)
Supplement: Supplementary file 1 — Supplementary Material 1. [file 40981_2026_859_MOESM1_ESM.docx]

Dear Dr. Obara and Reviewers,

We sincerely thank you for your careful evaluation of our manuscript entitled “Long-Term Pulmonary Function After Intrathoracic Versus Extrathoracic LVAD Implantation: Perioperative Implications at One Year” (JARP-D-26-00081).

We are grateful for the insightful and constructive comments, which have helped us improve the clarity, methodological rigor, and overall interpretation of our study. In response to the reviewers’ suggestions, we have revised the manuscript accordingly. The key revisions include:

(i) clarification of covariate selection using a directed acyclic graph (DAG),

(ii) modification of the mixed-effects model to avoid inclusion of baseline pulmonary function as a covariate in the primary analysis,

(iii) adjustment for surgical era to address potential temporal confounding, and

(iv) refinement of the Discussion to better reflect the limitations in causal interpretation and perioperative implications.

All changes have been highlighted in red in the revised manuscript.

A detailed point-by-point response to each comment is provided below.

We hope that the revised manuscript adequately addresses the reviewers’ concerns and would be suitable for publication in JA Clinical Reports.

**Reviewer #1 comment 1:**

More detailed patient characteristics should be provided in Table 1.

**Response:**

We thank the reviewer for this important suggestion. We have expanded Table 1 to include additional clinically relevant baseline characteristics, including underlying cardiac etiology and comorbidities, to improve the interpretability of the cohort and contextualize the observed pulmonary function changes.

**Related revised manuscript text:**

Table 1 (see below)

**Manuscript location:**

Line 154, Table 1. (Line 310)

|  | **Overall** | **Intrathoracic (HM3)** | **Extrathoracic (HMII)** | **P** |
| --- | --- | --- | --- | --- |
| Number | 190 | 76 | 114 |  |
| Age, years | 47 [37, 57] | 50 [40, 59] | 45 [36, 55] | 0.093 |
| Female sex, n (%) | 58 (30.5%) | 25 (32.9%) | 33 (28.9%) | 0.563 |
| Height, cm | 166.9 [159.9, 172.8] | 166.1 [159.0, 172.4] | 167.7 [160.5, 173.0] | 0.537 |
| Weight, kg | 56.6 [48.0, 69.0] | 55.4 [46.1, 68.1] | 57.9 [48.7, 69.5] | 0.196 |
| Body surface area, m^2^ | 1.64 [1.48, 1.81] | 1.62 [1.44, 1.78] | 1.65 [1.52, 1.83] | 0.200 |
| Underlying disease, n (%) |  |  |  | 0.700 |
| DCM | 91 (51.4%) | 35 (46.7%) | 56 (54.9%) |  |
| ICM | 23 (13.0%) | 11 (14.7%) | 12 (11.8%) |  |
| HCM | 27 (15.3%) | 12 (16.0%) | 15 (14.7%) |  |
| Myocarditis | 9 (5.1%) | 3 (4.0%) | 6 (5.9%) |  |
| Others | 40 (21.1%) | 15 (19.7%) | 25 (21.9%) |  |
| Surgical era | 2018 [2016, 2020] | 2021 [2020, 2022] | 2017 [2015, 2017] | <0.001 |
| Hypertension, n (%) | 11 (5.8%) | 1 (1.3%) | 10 (8.8%) | 1.000 |
| Preoperative Alb, g/dL | 3.7 [3.2, 4.0] | 3.6 [3.3, 4.0] | 3.7 [3.2, 4.0] | 0.740 |
| Preoperative Cr, mg/dL | 0.96 [0.81, 1.24] | 0.96 [0.76, 1.22] | 0.96 [0.82, 1.29] | 0.596 |
| Preoperative BUN, mg/dL | 18 [14, 24] | 18 [14, 22] | 19 [14, 26] | 0.227 |
| Preoperative T-Bil, mg/dL | 0.9 [0.6, 1.4] | 0.9 [0.7, 1.3] | 0.9 [0.6, 1.7] | 0.503 |
| Preoperative BNP, pg/dL | 579 [299, 1095] | 604 [293, 1160] | 575 [338, 1038] | 0.950 |
| Diabetes, n (%) | 34 (17.9%) | 3 (3.9%) | 31 (27.2%) | 0.344 |
| Smoking history, n (%) | 61 (32.1%) | 4 (5.3%) | 57 (50.0%) | 0.172 |
| Preoperative FVC, L | 3.02 [2.40, 3.46] | 2.90 [2.22, 3.32] | 3.07 [2.52, 3.49] | 0.066 |
| Preoperative %FVC | 88.0 [75.5, 99.5] | 84.7 [75.9, 97.4] | 90.4 [75.5, 102.6] | 0.266 |
| Preoperative FEV_1_, L | 2.35 [1.86, 2.83] | 2.16 [1.64, 2.74] | 2.39 [1.97, 2.84] | 0.030 |
| Preoperative %FEV_1_ | 79.0 [74.3, 83.8] | 78.0 [73.4, 82.3] | 80.0 [76.2, 84.0] | 0.113 |

**Reviewer #1 comment 2:**

Provide a DAG to justify covariate selection.

**Response:**

We agree that a causal framework is essential to justify covariate selection. We constructed a directed acyclic graph (DAG) to represent the assumed relationships among LVAD configuration, temporal factors, patient characteristics, and pulmonary function outcomes. This DAG informed our identification of confounders included in the primary models. The DAG is now provided as a supplementary figure.

**Related revised manuscript text:**

To inform covariate selection, a directed acyclic graph was constructed to represent the assumed relationships among LVAD configuration, temporal factors, patient characteristics, and pulmonary function outcomes. Based on this framework, age, sex, body surface area, and surgical era were identified as potential confounders and included in the primary models. (The directed acyclic graph is presented in Supplementary Figure S1.)

**Manuscript location:**

Lines 125–129, Supplementary Figure S1. (Page 32)

**Reviewer #1 comment 3:**

Temporal confounding due to different eras should be considered.

**Response:**

We appreciate this important point. Differences in implantation era between device groups represent a key source of potential confounding. Based on the DAG framework, we explicitly incorporated surgical era as a covariate in the primary mixed-effects models to account for temporal changes in indications, surgical techniques, and perioperative care. In addition, we have clarified in the Discussion that residual confounding may still remain despite adjustment.

**Related revised manuscript text:**

For each outcome, a mixed-effects model including time, pump configuration (intrathoracic vs extrathoracic), and their interaction term was fitted. The primary models adjusted for age, sex, body surface area, and surgical era to account for potential temporal confounding.

Importantly, the comparison between intrathoracic and extrathoracic configurations inherently reflects differences not only in pump position but also in device generation, implantation era, and perioperative management. Although we adjusted for surgery year in the primary analysis to account for temporal confounding, residual confounding related to changes in patient selection, surgical techniques, and perioperative care cannot be fully excluded. Therefore, the observed associations should not be interpreted as purely reflecting the mechanical effect of pump location alone. In line with this interpretation, smaller body size was a strong and independent predictor of lower postoperative pulmonary function, with lower body surface area consistently associated with lower FVC, %FVC, and FEV₁ across all adjusted models. Although female sex has been linked to smaller thoracic dimensions in prior studies (8), in our analysis, sex was associated with lower adjusted pulmonary function values, but this association appeared to be largely explained by body size, as reflected by body surface area. This likely reflects collinearity between sex and body size. Importantly, body surface area may more directly capture individual thoracic spatial constraints relevant to intrathoracic pump placement, providing a more physiologically meaningful metric than sex alone.

**Manuscript location:**

Lines 129–132.

Lines 191–205.

**Reviewer #1 comment 4:**

Including baseline pulmonary function as a covariate may introduce bias.

**Response:**

We thank the reviewer for highlighting this methodological concern. In the revised analysis, preoperative pulmonary function values were removed from the primary mixed-effects models because they are part of the longitudinal outcome vector and may lead to endogenous regression. Instead, baseline-adjusted models were conducted as supplementary analyses to confirm robustness.

**Related revised manuscript text:**

Preoperative pulmonary function values were not included as covariates in the primary models because they are part of the longitudinal outcome vector and were therefore not treated as confounders. Instead, baseline-adjusted models were performed as supplementary analyses. (Supplementary Figure S2.)

**Manuscript location:**

Lines 133–136.

**Reviewer #2 comment 1:**

Clinical relevance for anesthesiology practice should be clarified.

**Response:**

We appreciate this insightful comment. We have strengthened the Discussion to clarify the clinical implications for contemporary anesthetic practice. Although current LVAD implantation predominantly involves HeartMate 3, the increasing number of LVAD recipients has led to a growing population of patients with previously implanted HeartMate II devices undergoing non-cardiac surgery. Therefore, understanding device-related differences in pulmonary function remains clinically relevant. We also emphasized that patients with smaller body size or limited pulmonary reserve may require more cautious perioperative respiratory management.

**Related revised manuscript text:**

From a perioperative perspective, restrictive ventilatory changes following LVAD implantation may influence postoperative respiratory management, ventilator weaning strategies, and long-term functional recovery. However, because the present study did not directly assess perioperative outcomes such as duration of mechanical ventilation, reintubation, or ICU stay, these interpretations should be considered hypothesis-generating rather than definitive evidence of perioperative impact. Taken together, our findings highlight the potential importance of considering patient-specific thoracic dimensions when selecting LVAD configurations and underscore the need for further studies integrating anatomical, physiological, and clinical outcome data to better understand the respiratory consequences of LVAD implantation.

**Manuscript location:**

Lines 221–229.

**Reviewer #2 comment 2:**

Device differences (era, generation) limit causal interpretation.

**Response:**

We fully agree with this important concern. We explicitly incorporated surgical era as a covariate in the primary mixed-effects models to account for temporal changes in indications, surgical techniques, and perioperative care. In addition, we have revised the manuscript to explicitly acknowledge that the comparison between intrathoracic and extrathoracic configurations inherently reflects differences in device generation, implantation era, and perioperative management. We have softened causal language and clarified that our findings should not be interpreted as purely reflecting the mechanical effect of pump location. In addition, we have revised the manuscript to consistently soften causal language throughout, ensuring that the findings are interpreted as associations rather than direct causal effects.

**Related revised manuscript text:**

For each outcome, a mixed-effects model including time, pump configuration (intrathoracic vs extrathoracic), and their interaction term was fitted. The primary models adjusted for age, sex, body surface area, and surgical era to account for potential temporal confounding.

Importantly, the comparison between intrathoracic and extrathoracic configurations inherently reflects differences not only in pump position but also in device generation, implantation era, and perioperative management. Although we adjusted for surgery year in the primary analysis to account for temporal confounding, residual confounding related to changes in patient selection, surgical techniques, and perioperative care cannot be fully excluded. Therefore, the observed associations should not be interpreted as purely reflecting the mechanical effect of pump location alone. In line with this interpretation, smaller body size was a strong and independent predictor of lower postoperative pulmonary function, with lower body surface area consistently associated with lower FVC, %FVC, and FEV₁ across all adjusted models. Although female sex has been linked to smaller thoracic dimensions in prior studies (8), in our analysis, sex was associated with lower adjusted pulmonary function values, but this association appeared to be largely explained by body size, as reflected by body surface area. This likely reflects collinearity between sex and body size. Importantly, body surface area may more directly capture individual thoracic spatial constraints relevant to intrathoracic pump placement, providing a more physiologically meaningful metric than sex alone.

**Manuscript location:**

Lines 129–132.

Lines 191–205.

**Reviewer #2 comment 3:**

Perioperative implications are indirect due to lack of clinical outcomes.

**Response:**

We agree that perioperative implications should be interpreted cautiously given the absence of direct clinical endpoints. We have revised the Discussion to clearly state that our findings are hypothesis-generating and not definitive evidence of perioperative impact. We believe that, despite the predominance of HeartMate 3, patients with previously implanted LVADs, including HeartMate II, continue to present for non-cardiac surgery, making these findings clinically meaningful.

**Related revised manuscript text:**

From a perioperative perspective, restrictive ventilatory changes following LVAD implantation may influence postoperative respiratory management, ventilator weaning strategies, and long-term functional recovery. However, because the present study did not directly assess perioperative outcomes such as duration of mechanical ventilation, reintubation, or ICU stay, these interpretations should be considered hypothesis-generating rather than definitive evidence of perioperative impact. Taken together, our findings highlight the potential importance of considering patient-specific thoracic dimensions when selecting LVAD configurations and underscore the need for further studies integrating anatomical, physiological, and clinical outcome data to better understand the respiratory consequences of LVAD implantation.

**Manuscript location:**

Lines 221–229.

**Reviewer #2 comment 4:**

Selection bias due to inclusion of only 1-year survivors.

**Response:**

We thank the reviewer for this important observation. We have explicitly acknowledged the potential for survivor bias and clarified that the findings may not be generalizable to more severely ill patients who did not survive to 1 year or were unable to undergo pulmonary function testing.

**Related revised manuscript text:**

Sixth, the analysis included only patients who survived 1 year and underwent pulmonary function testing, which may introduce survivor bias and limit the generalizability of the findings to more severely ill populations.

**Manuscript location:**

Lines 249–251.
